# Complexation of β‐lactoglobulin with gum arabic: Effect of heat treatment and enhanced encapsulation efficiency

**DOI:** 10.1002/fsn3.2103

**Published:** 2021-01-18

**Authors:** Mengna Cao, Jian Gao, Yang Li, Chengzhi Liu, Jieyu Shi, Fangfang Ni, Gerui Ren, Hujun Xie

**Affiliations:** ^1^ School of Food Science and Biotechnology Zhejiang Gongshang University Hangzhou China

**Keywords:** composite particles, encapsulation, gum arabic, heat treatment, interaction, β‐lactoglobulin

## Abstract

Heat treatment is widely used in food industry. Proteins and polysaccharides as important natural polymers in food, under heat treatment, the interactions between them could mediate the conformation and functional properties of proteins. Thermally induced β‐lactoglobulin‐gum arabic complexes (β‐Lg‐GA) were fabricated, and the effect of heat treatment on physicochemical properties of the complexes was systematically investigated. The average particle size of β‐Lg‐GA complexes decreased with temperature increased, at 85°C, a smaller size of 273 nm was obtained. A saturated adsorption of GA was found when mass ratio of β‐Lg/GA was <1:2. At pH = 4.2–7.0, electrostatic attraction between β‐Lg and GA was low and a fairly constant turbidity was observed, the formed composite particles had good stability to the pH value. Through UV, fluorescence, and FTIR spectroscopy, it was found that formation of the nanoparticles relied on thermal denaturation and aggregation of protein, the electrostatic, hydrophobic, and hydrogen bonding interactions between β‐Lg and GA were also important. Scanning electron microscope further indicated β‐Lg and GA had good compatibility, and the complexes had a spherical core–shell structure at molecular level. In addition, these prepared natural nanoparticles by heat treatment show significantly higher encapsulation efficiency for (‐)‐epigallocatechin‐3‐gallate (EGCG) than that of unheated, thus could be used as a promising carrier for biologically active substances.

## Graphical abstract

Novel thermally induced β‐lactoglobulin‐gum arabic complexes (β‐Lg‐GA) were fabricated. A preferable size of 273 nm was obtained at 85°C. And our findings indicated that heating not only denatured β‐Lg, but also promoted structural rearrangement of β‐Lg and GA to form a more stable core‐shell structure. Moreover these prepared natural nanoparticles by heat treatment show significantly higher encapsulation efficiency for (‐)‐epigallocatechin‐3‐gallate (EGCG) than that of unheated, thus could be used as a promising carrier for biologically active substances.

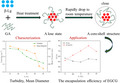

## 1 INTRODUCTION

The β‐lactoglobulin (β‐Lg) is the chief protein fraction of milk proteins. Due to easy availability and the important economic role of milk, β‐Lg was widely investigated in the basic protein science field over the past decades (Hansted et al., 2011; Moro et al., 2011). The β‐Lg belongs to globular protein with molecular weight of 18.3 kDa. It contains 162 amino acids with two intramolecular disulfide bonds and a free sulfhydryl (Hoffmann & van Mil, 1997; Hoffmann et al., 1997). The thermal denaturation temperature of β‐Lg is about 70°C at pH 7. When induced by heating, the functional properties of β‐Lg are easily modified and the conformation also can be transformed. Native β‐Lg becomes unfolded, disulfide bonds and hydrophobic groups which are always in the protein interior are exposed to outside, thus resulting in protein molecules aggregation through chemical (inter‐ and intramolecular disulfide interchange) and physical (hydrophobic, electrostatic, hydrogen, and van der Waals forces) interactions (Delahaije et al., 2015; Ikeda et al., 1999; Weijers et al., 2005). However, the nature of thermally induced aggregation depends on many factors such as solution pH, ionic composition, ionic strength, and heating conditions, and the mechanism is very complex (Hoffmann & van Mil, 1999; Le Bon et al., 1999; Verheul et al., 1998).

Polysaccharide, as another significant biopolymer in food, also has an important influence on the thermal aggregation of proteins. Protein‐polysaccharide particles prepared through heat treatment have good stability that could overcome the dissociation of unheated protein–polysaccharide particles when the environmental conditions were altered (Hong & McClements, 2007; Kelly et al., 1994; Sanchez & Paquin, 1997; Schmitt et al., 1998; Yu et al., 2006). Jones and McClements (Jones et al., 2009a, 2009b; 2008) made an in‐depth study of the β‐Lg‐polysaccharide system created by heat treatment. Stable polymer particles can be prepared by heating the protein and different anionic polysaccharides at a temperature higher than the denaturation temperature of the protein. Su et al. (2020) have fabricated a superior delivery system of lutein through gum arabic and heated β‐Lg and used it to form stable Pickering emulsions. After 12 weeks of preservation, 91.1% of lutein remained in the emulsion which containing 70% oil phase. The properties of the formed biopolymer particles were closely related to the types of polysaccharides. The interaction between protein molecules and anionic polysaccharide chains was different, resulting in different particle behaviors.

These limited examples showed that polysaccharides with electrostatic attraction played an important role in protein aggregation. However, heat treatment was usually employed to denature protein before they interacted with polysaccharide. This might impair potential of the proteins as valuable bioactive substances. In addition, heat treatment is widely used in food industry. So it is necessary to study the interactions between protein and polysaccharide together induced by heating.

Gum arabic (GA) is an amphiphilic polysaccharide, has widespread applications in food industry, because of its persistent stability in various extreme environments and excellent functional properties such as low cost, low viscosity, high solubility, emulsification, and negatively charged properties. In recent years, GA has been combined to a variety of proteins, such as milk, egg, wheat, rice, and soybean proteins (Li et al., 2019; Niu et al., 2017; Wei & Huang, 2019). The latest research showed that the electrostatic interactions were the main driven force for the formation of the β‐Lg‐GA nanoparticles (Wang et al., 2020). Schmitt et al. (2000) studied the effect of protein aggregates on the complex coacervation between β‐Lg and GA at pH 4.2, which showed that the pH can affect β‐Lg aggregation and thus the formation of β‐Lg‐GA nanoparticles. GA also could improve the storage stability and antibacterial ability of β‐Lg stabilized d‐limonene emulsion at pH 4.0 based on electrostatic layer‐by‐layer deposition (Su, 2018). Moreover, it was found that the stability of β‐Lg‐GA nanocomplexes increased with the increase of GA concentration by Hosseini et al. (2016).

In the present work, GA is used as a template of polysaccharide, and the effect of thermally induction on the behavior of β‐Lg‐GA complex systems has been examined. The factors such as temperature, heating time, pH, concentration, ionic composition, and ionic strength that affecting the preparation and physicochemical properties of the complexes under thermal condition are systematically investigated. In addition, a preliminary study and exploration about the molecular interactions between protein and polysaccharide induced by heat treatment are undertaken through ultraviolet spectroscopy, fluorescent, Fourier transform infrared spectroscopy, and scanning electron microscope. Furthermore, the fabricated complexes have been evaluated as a new delivery system for (‐)‐epigallocatechin‐3‐gallate (EGCG).

## 2 MATERIALS AND METHODS

### 2.1 Materials

Gum arabic (GA), β‐lactoglobulin (β‐Lg, purity ≥90%), epigallocatechin‐3‐gallate (EGCG), NaCl, BaCl_2,_ sodium dodecyl sulfate (SDS), urea, NaOH, and all other reagents were bought from Aladdin reagent. HCl (purity = 36%–38%) was bought from Hangzhou Shuanglin Chemical Reagent Factory, and deionized water was selected for experiments.

### 2.2 Preparation of β‐Lg‐GA composite solutions

β‐LG and GA were dissolved in deionized water with pH value of 7.0 and stirred at 500 rpm for 4 hr at room temperature, separately. Then, placed at 4°C overnight to be fully hydrated and dissolved. The solutions of β‐Lg and GA were mixed under different concentrations or ratios and placed for 24 hr. Then, the mixed β‐Lg‐GA samples were heated in a 50–90°C water bath for 0–30 min. Samples were taken out and shaken rapidly for 1 min, chilled in a cold water bath for 2 hr, and refrigerated at 4°C for 12 hr. The pH of the solutions was adjusted with different concentrations of NaOH or NaCl (0.05, 0.1, 0.25, 0.5, 1, 2 mol/L). In general, total concentration of β‐Lg‐GA composite solution was 0.05 wt% (mass ratio of β‐Lg/GA = 2:1). However, different β‐Lg/GA mass ratios (from 1:3 to 3:1) and total concentrations (from 0.025 to 0.1 wt%) of β‐Lg‐GA composite solutions were also studied.

### 2.3 Preparation of β‐Lg‐GA nanoparticles

The mixed solution (mass ratio of β‐Lg/GA = 2:1) was heated (85°C) at pH 4.2 for 20 min and centrifuged at 10,000 r/min for 15 min. The concentration of the complex was kept in the mixed solution at 1 wt%. The lower phase was collected and dried in a freeze dryer for 48 hr.

### 2.4 Turbidity measurement

The turbidity of the β‐Lg‐GA complex solution at different conditions was investigated by UV/vis spectroscopy (UV‐2600, Shimadzu) at 600 nm.

### 2.5 Determination of particle size

The mean diameter of the heat‐treated β‐Lg solution, GA solution, and β‐Lg‐GA composite solutions was measured by a Nano‐ZS type laser particle size analyzer (Zeta Nano‐ZS, Malvern Instruments), respectively. All experiments were performed at 25°C. Each sample was measured 12 times in parallel three times, and the average value was taken. The material was protein with the refractive index of 1.45, and the dispersant was water with the refractive index of 1.33.

### 2.6 Ultraviolet spectral measurements (UV)

The concentration of β‐Lg‐GA composite solution was 0.05 wt%, and the mass ratio of β‐Lg/GA was maintained at 2:1 in the process of heat treatment. The spectra of samples were detected by UV‐2600 (Shimadzu) in the scanning range of 200–600 nm.

### 2.7 Fluorescence spectroscopy

The concentration of β‐Lg‐GA composite solution was 0.05 wt%, and the mass ratio of β‐Lg/GA was maintained at 2:1 in the process of heat treatment. The samples were measured by fluorescence spectrophotometer (RF‐5301PC, Japan). The experimental parameters were set as: (a) Endogenous fluorescence, excitation light wavelength was 290 nm, and the scanning range was 220–400 nm, (b) synchronous fluorescence, the difference between excitation light wavelength and scanning start wavelength was 15 nm and 60 nm, respectively, and excitation wavelength was 220 nm.

### 2.8 Fourier transform infrared spectroscopy (FTIR)

The particles of β‐Lg‐GA and KBr were mixed at the ratio of 1:100 and were pressed to disks. The FTIR spectra were tested in the wavenumber range of 800 − 4,000 cm^−1^ by Fourier transform spectrophotometer (Nicolet iS5, Thermo Fisher).

### 2.9 Microscopic morphology (SEM)

The surface morphology of β‐Lg‐GA nanoparticles was observed by scanning electron microscope (*SEM*, SU8010, Hitachi) at a working voltage of 10.0 kV.

### 2.10 Encapsulation efficiency (EE)

The mixed solution of β‐LG and GA was heated at different temperatures for 20 min and then rapidly mixed with EGCG solution (mass ratio of β‐Lg/GA = 2:1; content of EGCG was 0.2 mg/ml). The mixture was shaken in a vortex oscillator for 5 min, immersed in a cold water bath for 3 hr to make the solution fully react, and then centrifugated at 8,000 r/min at 4°C for 30 min. The β‐LG‐EGCG was prepared as the above method without GA. The content of free EGCG in the supernatant was determined by high‐performance liquid chromatography (HPLC, Waters e2695).

HPLC conditions: C18 column, 20 μl injection volume, 1.0 ml/min flow rate. The gradient elution of mobile phase (A: 80% methanol + 20% water + 0.5% acetic acid, B: 90% water + 10% methanol + 0.5% Acetic acid) was from 20:80 to 80:20 with 22 min analysis time. The detection wavelength was 274 nm.

The EE values were calculated according to the following equation.

EE(%)=[(Total EGCG‐Free EGCG)/Total EGCG]×100%

### 2.11 Statistical analysis

Each sample was measured three times in parallel, and the average and standard deviation were taken. One‐way ANOVA analysis was carried out on the data using SPSS 18.0 software, and the significance level was 5%.

## 3 RESULTS AND DISCUSSION

### 3.1 Heating temperature

Turbidity is an effective method to characterize the intermolecular binding (Gao et al., 1997; de Kruif & Tuinier, 2001; Zorilla et al., 2011); thus, some preliminary experiments were test by turbidity. It was found that turbidity of the mixed system of β‐Lg and GA remained relatively low at pH = 4.8–7, when pH <4.8, the mixed system formed a large aggregation, so initially the pH = 4.5–6 was selected as a suitable pH range to study the various factors that influence the formation of β‐Lg‐GA polymer particles.

**FIGURE 1:**
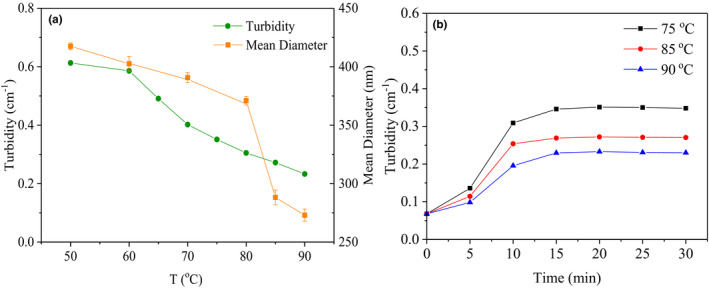
Effect of different heating temperatures (50–90°C) and heating times on the turbidity and particle size of β‐Lg‐GA complex at pH 4.5. The concentration of β‐Lg‐GA mixture solution was 0.05 wt%; the mass ratio of β‐Lg and GA was 2:1

The effect of heating temperatures was firstly studied (Figure 1a). Turbidity of the heating samples decreased when temperature increased from 50°C to 90°C, and there was no precipitation observed. The average particle size of the polymers decreased from 417 nm to 273 nm as the temperature increased, and the polymer particles had a polydispersity index between 0.5 and 0.6, which consistent with heating the β‐Lg solution alone. It is probably because the protein structure rapidly folds and aggregates at higher temperatures. Thus, abundant nuclei are generated, which cannot grow up to larger particles before all the protein molecules are assembled. Under heat treatment, partial proteins might be separated from polysaccharides, after that free proteins accumulate in the aqueous phase, β‐Lg and GA combine in a loose state. When the temperature drops rapidly to room temperature, the energy of system decreases, β‐Lg and GA form a close core–shell structure, the core composed by β‐Lg aggregates, and GA wrapped in the outer layer. Hence, the particle sizes change smaller. The similar change in particle size during the heat treatment of β‐Lg and pectin has reported by Jones et al. (Jones et al., 2009b; Jones & McClements, 2008).

### 3.2 Heating time

The effect of heating time was shown in Figure 1b. Samples were heated at 75, 85, and 90°C with different times, respectively. Turbidity of samples gradually increased from 0 to 15 min, then basically smooth. Average particle size of the samples also increased during the first 15 min, but decreased slightly during the next 15 min (Figure S1). Hoffmann et al. (2009) observed particle sizes of aggregated state generated by the spherical protein solution increased as heat treatment times in a certain time range. The results may indicate the complex formed by β‐Lg‐GA has a similar aggregation behavior with the globulin denaturation at higher temperatures.

### 3.3 Concentration and ratio of β‐Lg/GA

**FIGURE 2:**
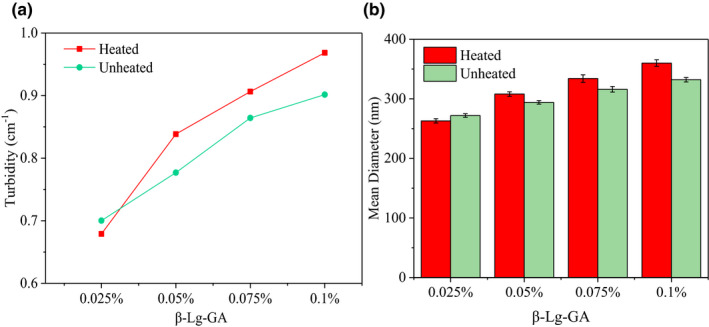
The turbidity (a) and particle size (b) of different concentrations of β‐Lg‐GA solution at pH 4.5. The mass ratio of β‐Lg and GA was 2:1

Thirdly, the influence of β‐Lg‐GA solution concentration on the formation of heat‐induced protein–polysaccharide complexes was tested. It could be seen from Figure 2a that turbidity increased as concentration of β‐Lg‐GA solution increased from 0.025 wt% to 0.1 wt%. The β‐Lg molecule denaturated or structural rearranged during heating, so that the turbidity of the heat‐treated composite solution was greater than that of the unheated. The particle size of β‐Lg‐GA complex through heat treatment and nonheat treatment (Figure 2b) both increased as the increase of composite solution concentration, and the particle size of heated complex bigger than that of unheated, which had the same trend with the turbidity experiments in Figure 2a, indicating that certain changes in protein molecules occurred during heating.

**FIGURE 3:**
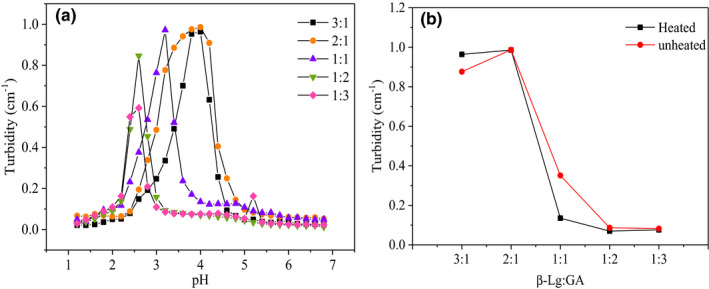
(a) The turbidity of different mass ratios of β‐Lg/GA composite solution with pH. (b) The turbidity of different mass ratios of β‐Lg‐GA mixture solution before and after heat treatment at pH 4.5. The concentration of β‐Lg‐GA mixture solution was 0.05 wt% heated at 85°C for 20 min

The concentration of polysaccharide plays important role in the generation of heated protein–polysaccharide particles. Here we took the mass ratio of polysaccharide to protein as the research object. The turbidity of β‐Lg‐GA composite solutions with various mass ratios of β‐Lg/GA was shown in Figure 3a,b. With the increase of GA contents, the pH of the maximum turbidity always moved to a lower pH value, might due to the electrostatic repulsion between the negative charge on the side chain of the GA offsetting a portion of the electrostatic attraction between GA and β‐Lg, therefore a lower pH was required to enhance the electropositivity of protein, thus improve the electrostatic attraction of protein and polysaccharide (Chen et al., 2007, 2014; Xie et al., 2019). The turbidity decreased sharply when the mass ratio of β‐Lg/GA changed from 2:1 to 1:2 both before and after heat treatment. The formation of β‐Lg‐GA composite solutions was greatly affected by the content of GA. When the low level of GA (β‐Lg/GA = 3:1, 2:1) was added, the large aggregate was formed. It was probably because the charge neutralization and the bridging flocculation caused by the adsorption of anionic GA onto β‐lg aggregates (Santipanichwong et al., 2008; Su et al., 2020). It was worth mentioning that whether the heated or unheated system, when the mass ratios of β‐Lg/GA were 1:2 and 1:3, the turbidity became the minimum and stable, which indicated that GA has been saturated by adsorption; thus, the β‐Lg/GA complexes by heat treatment had a core–shell structure. The similar findings were reported by Su (Su et al., 2020). They found while mass ratios of β‐Lg/GA were 1:2, 1:3, and 1:5, zeta potential values of the complexes were near the zeta potential value of GA alone.

### 3.4 Effect of pH

**FIGURE 4:**
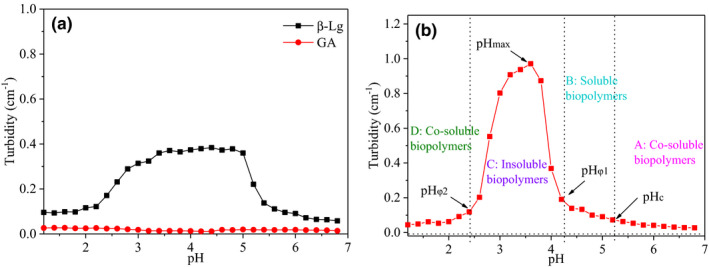
(a) The turbidity of individual β‐Lg and GA at different pH. (b) The turbidity of β‐Lg‐GA solution at different pH. The concentration of β‐Lg and GA solution was 0.025 wt%, and the concentration of β‐Lg‐GA mixture solution was 0.05 wt%; the mass ratio of β‐Lg and GA was 2:1. The solutions were all heated at 85°C for 20 min

It is well known that protein is greatly affected by pH value. The purpose of this experiment is to explore the interaction between protein and polysaccharide under heating conditions by adjusting pH and then prepare complexes based on this. The turbidity of individual (Figure 4a) and mixed (Figure 4b) biopolymer solutions was measured.

As shown in Figure 4a, in the whole pH range, the turbidity of GA solution changed little, approach to zero, demonstrating that there are no aggregates generated by GA with strong dynamic light scattering absorption. The aqueous solution of GA is a negatively charged polyanion, and the strong electrostatic repulsion between GA would prevent them to form aggregates. While the turbidity of β‐Lg solutions was near zero at pH = 1.0–2.5 and pH = 5.5–7.0, the maximum turbidity was about 0.38 ± 0.003 in a wide range of pH = 5.5–2.5, which indicated that it was self‐aggregating not only around the isoelectric point (pH = 5.1–5.3) of the heat‐treated β‐Lg solution.

As shown in Figure 4b, the process was divided into four regions according to the form of the complex, A: co‐soluble biopolymers; B: soluble biopolymers; C: insoluble biopolymers; D: co‐soluble biopolymers (Xie et al., 2019). There were four special pH values at the boundary points of these four regions, pH_c_ was about 5.2, which was the starting point for the formation of soluble complexes; pH_φ1_ was about 4.2, which meant that insoluble complexes begin to appear; pH_φ2_ was about 2.4, which represented that the insoluble complex dissociates completely; between pH_φ1_ and pH_φ2_, there was a turbidity maximum, and the corresponding pH_max_ at this time was about 3.6. In contrast to β‐Lg alone, the turbidity of β‐Lg‐GA complex had changed significantly, and the maximum turbidity of the complex had moved to a lower pH, indicating that there was an electrostatic interaction between GA and β‐Lg, and GA had a great influence on the formation of the heat‐induced protein–polysaccharide complex. Therefore, both thermal denaturation and aggregation of proteins and the electrostatic interaction of proteins and polysaccharides play key role in the preparation of composite particles (Hu et al., 2016; Hu et al., 2016).

### 3.5 Effect of salt ion strength and type

**FIGURE 5:**
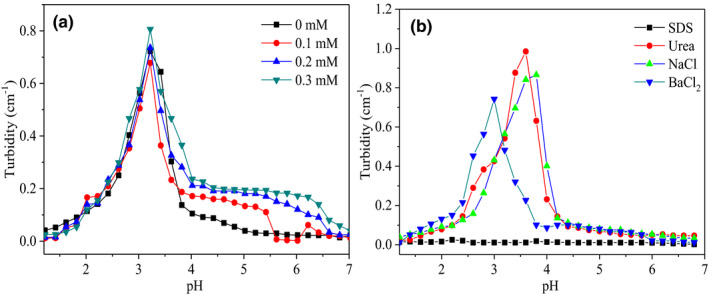
The turbidity of β‐Lg‐GA mixture solution with different salt ion concentrations (a) and different salts (b) after heat treatment. The total concentrations of sodium chloride were 0, 0.1, 0.2, and 0.3 mM, respectively. The concentration of SDS, urea, and NaCl is 10 mmol/L, respectively. The concentration of BaCl_2_ is 5 mmol/L. The concentration of β‐Lg‐GA mixture solution is 0.05 wt%, and the mass ratio of β‐Lg and GA is 2:1

The influence of the salt ion strength on turbidity of thermally induced β‐Lg‐GA samples was also examined (Figure 5a). It was showed that turbidity of β‐Lg‐GA complex solution increased first and then decreased as the pH decreased, which was similar to the microgel formed by heat treatment of β‐Lg and pectin reported by Jones et al (Jones et al., 2009b; Verheul et al., 1998). The pH_φ2_ was the complete end point of the insoluble complex dissociation which did not move with the addition of NaCl. Under different NaCl concentrations, the trend of turbidity changes of the β‐Lg‐GA complex solution with certain pH values was almost the same. It was speculated that this might be due to the fact that NaCl only shielded the charge on the surface of β‐Lg‐GA polymer particles, but did not have much influence on the electrostatic interaction inside the complex. This might be ascribed to the outer layer of the complex was coated by GA molecules. This phenomenon supported the heat‐induced β‐Lg‐GA particles had a core–shell structure.

To investigate the effect of solution ionic composition on the generation of the heat‐treated β‐Lg‐GA complexes, SDS, urea, NaCl, and BaCl_2_ were chosen as salt ion species (Figure 5b). It was noteworthy that SDS could destroy the hydrophobic interaction and electrostatic interaction between polymers and urea could affect the hydrogen bond interaction between protein and polysaccharide (Chen et al., 2007, 2014). As showed in Figure 5b, SDS made the β‐Lg‐GA composite solution before and after heat treatment show a clear and transparent state, while urea had no obvious influence on the turbidity of the composite solution. The NaCl and BaCl_2,_ which could affect the electrostatic interaction between proteins and polysaccharides, were also added to β‐Lg‐GA composite solution, respectively. It was found, NaCl, BaCl_2_ made the pH_max_ of the heat‐treated β‐Lg‐GA composite solution shift to a lower pH value, and this was because Na^+^ and Ba^2+^ can neutralize the negative charge sites on the GA side chain. Due to the presence of steric hindrance, the remaining positive charge sites require a lower pH value to make the electrostatic interaction stronger than steric hindrance in order to form β‐Lg‐GA composite solution better, indicating that electrostatic interaction had a great influence on the formation of nanoparticles.

### 3.6 Heat‐induced molecular interactions between β‐Lg and GA

#### 3.6.1 UV spectrum analysis

**FIGURE 6:**
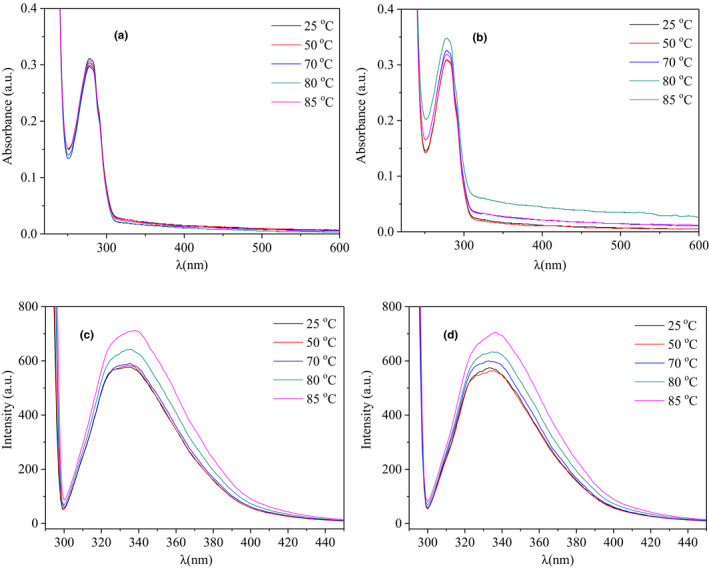
UV and fluorescence spectra under different temperatures (25–85°C) at pH = 7 for 20 min. (a) The UV spectra of β‐Lg solution; (b) the UV spectra of β‐Lg‐GA composite solution. (c) The fluorescence spectra of β‐Lg solutions; (d) the fluorescence spectra of β‐Lg‐GA mixture solutions

UV spectroscopy was used to have an insight into the impact of heat treatment on the interaction nature between β‐Lg and GA (Figure 6). All samples have an absorption peak at 280 nm, which may be due to the absorption of β‐Lg by tryptophan and tyrosine residues. For the β‐Lg‐GA composite system, whether heated or not, both had a higher absorbance than the β‐Lg solutions (compared Figure 6b–a), which suggested the present of GA might alter the structure of the β‐Lg, resulting more tryptophan and tyrosine residues exposed to the surrounding solvents.

#### 3.6.2 Fluorescence spectroscopy analysis

The interaction between protein and polysaccharide would change the fluorescence parameters, intensity, or quantum yield of tryptophan and tyrosine, so fluorescence spectra are commonly used to detect changes of protein spatial structure.

Endogenous fluorescence spectra and synchronous fluorescence spectra were both examined. Figure 6c,d showed the endogenous fluorescence spectra of β‐Lg solutions and β‐Lg‐GA composite solutions after heat treatment at 25–85°C. The fluorescence spectra of the pure β‐Lg solutions and β‐Lg‐GA composite solutions showed a slight red shift after heat treatment, indicating that conformation of protein had changed, resulting in the changes of tryptophan microenvironment of the β‐Lg molecule occurred. As the temperature increased, the fluorescence intensity of β‐Lg increased from 577 to 711 a.u. while β‐Lg‐GA composite solution increased from 575 to 705 a.u. The increase of β‐Lg‐GA composite solution was less than that of β‐Lg itself, demonstrating that the presence of GA improved the thermal stability of composite system (Liu et al., 2020).

At the same time, the effect of different temperatures (25–85°C) on the synchronous fluorescence (Δλ = 15 nm and Δλ = 60 nm) spectra was studied (Figure S2) and found that as the temperature increased, the fluorescence intensity increased to some extent. Fluorescence intensity of wavelength shift at 60 nm was significantly higher than the fluorescence intensity of wavelength shift at 15 nm, indicating that the microenvironment of tyrosine had little change, but the more hydrophobic tryptophan residues were exposed outside, the hydrophobicity of the complex was enhanced. This result was consistent with the endogenous fluorescence.

#### 3.6.3 Fourier transform infrared spectroscopy (FTIR)

**FIGURE 7:**
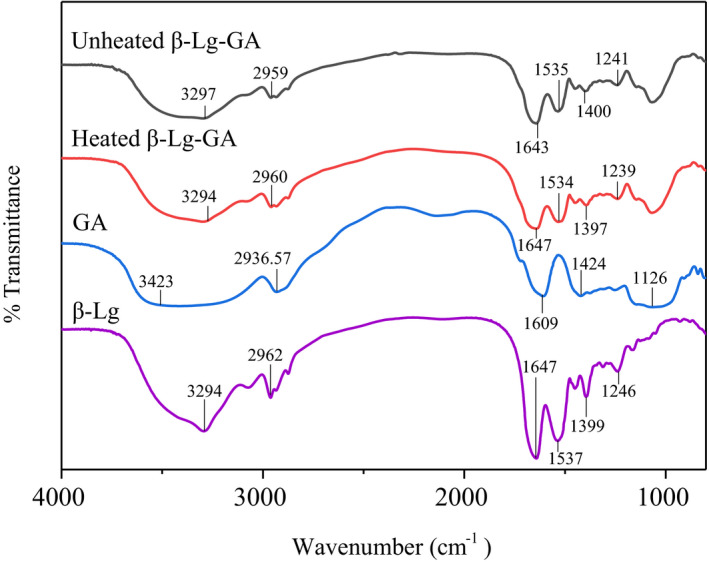
Fourier transform infrared spectroscopy spectra of β‐Lg, GA, β‐Lg‐GA, and heated β‐Lg‐GA complex

Fourier infrared spectroscopy could characterize the changes in the internal groups of molecules and also has potential to discuss the interactions between polymer particles. As shown in Figure 7, the range of 3100–3500 cm^−1^ is ascribed to the stretching vibration of O‐H or N‐H bond (Liang et al., 2015) and asymmetric stretching vibration absorption peak of ‐CH_2_ is around 2,960 cm^−1^. The peak of amide I band caused by stretching vibration absorption of C = O double bond is around 1643 cm^−1^, and 1535 cm^−1^ peak is the bending vibration of amide II band caused by N − H bond (Chang et al., 2017; Xiao et al., 2015). 1,400 and 1,241 cm^−1^ is the stretching vibration caused by C − N bond of primary and secondary amide, respectively (Verheul et al., 1998). Compared with the infrared spectrum of β‐Lg‐GA complex before and after heat treatment, the N‐H bond, amide I, and amide II bands shifted to a little degree, indicated the existence of hydrogen bonding and electrostatic interactions in the system. However, the hydrogen bonding maybe not the main interaction between β‐Lg and GA under heated treatment.

#### 3.6.4 Microscopic morphology

**FIGURE 8:**
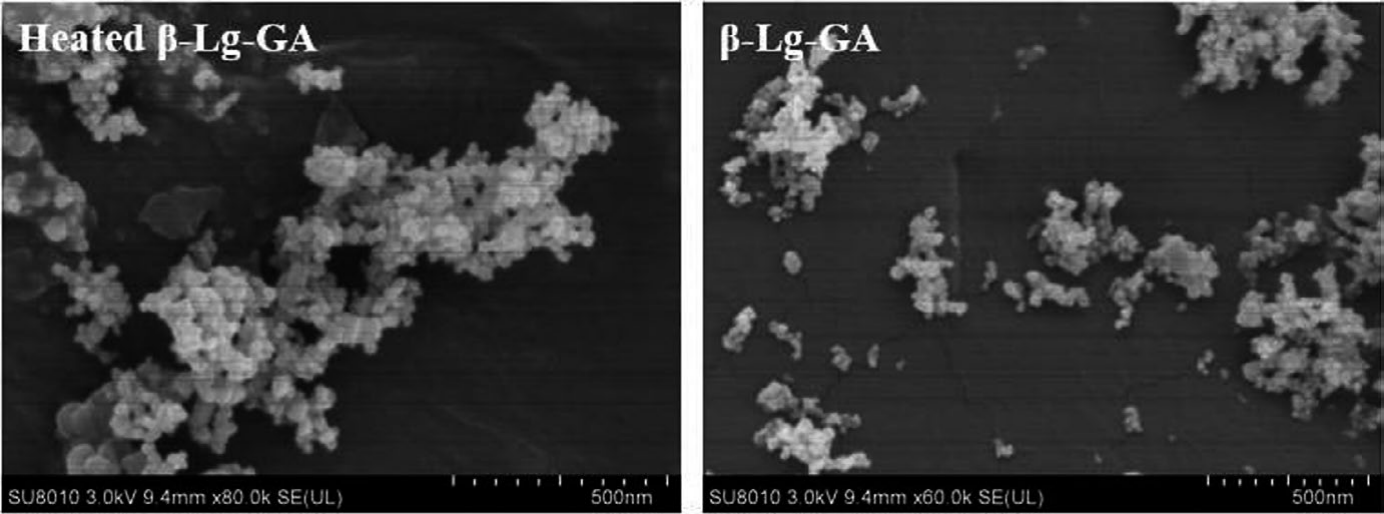
Scanning electron microscope images of β‐Lg‐GA nanoparticles before and after heat treatment

It was observed that in *SEM* images (Figure 8) the complex nanoparticles obtained before and after the heat treatment were both spherical particles, but the heat‐treated nanoparticles aggregated into larger nano‐clusters, which indicated that the heat treatment caused the β‐Lg‐GA nanoparticles to aggregate. The results of *SEM* had some differences compared with the particle size results measured by DLS. This was proposed to the inherent differences in particle size detection between DLS and *SEM*. DLS was used to determine the sizes of particles in the solution, while *SEM* was the image of particles in freeze‐dried powder. Moreover, it was difficult to identify individual particles clearly because the boundary of adjacent particles was fuzzy. This might be due to the GA molecules coated on surface of β‐Lg aggregations generated a membrane structure. The *SEM* results further supported β‐Lg and GA had good compatibility and had a core–shell structure at the molecular level.

### 3.7 Encapsulation efficiency

**FIGURE 9:**
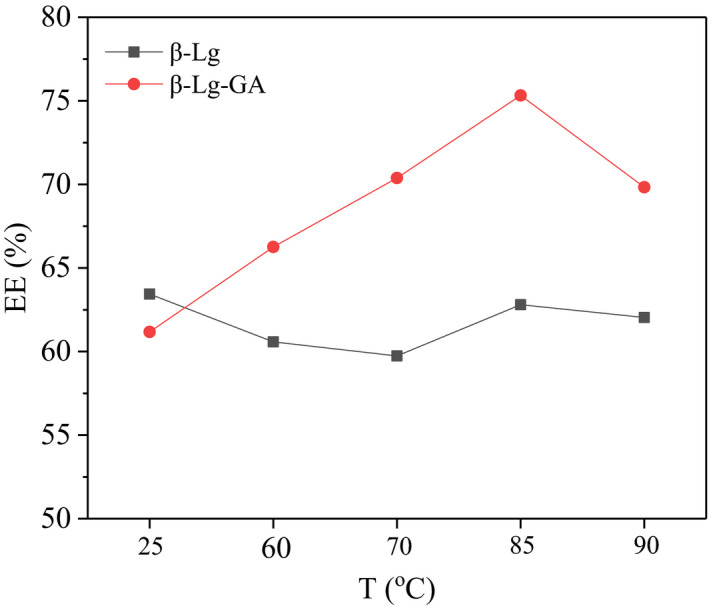
The influence of temperature on β‐Lg and β‐Lg‐GA nanoparticles encapsulated EGCG

The composite nanoparticles formed by heating protein and polysaccharide together may be very useful as delivery system to encapsulate functional components. EGCG, the main component of green tea polyphenols, is a catechin monomer isolated from tea. It can capture active oxygen and is used as a strong natural antioxidant in the food industry. It also has antibacterial, antitumor, and nervous system protection effects. In recent years, EGCG has become a focus of research for its remarkable health benefits. However, the stability of EGCG is not high. It is easily affected by factors such as pH, temperature, light, metal ions, and enzymes, which limits the application of EGCG in real life (Fujiki & Suganuma, 2012; Higdon & Frei, 2003; Sang et al., 2006; Singh et al., 2011). Hence, the encapsulation of EGCG by β‐Lg‐GA complexes was preliminarily evaluated. As shown in Figure 9, the EE value of EGCG in single β‐Lg nanoparticles was low and little affected by temperature. When EGCG was encapsulated in β‐Lg‐GA nanoparticles, the EE value was only 61% at room temperature. With the increase of temperature, the EE value increased significantly and reached the highest of 75% at 85°C. After heat treatment, β‐Lg‐GA nanoparticles have improved encapsulation efficiency on EGCG, which can be used as promising carrier materials.

## 4 CONCLUSION

In this work, the β‐Lg‐GA composite system was formed via controlling the temperature of heat treatment, and the behavior of polymer particles was regulated by mediating heating time, temperature, pH, concentration, ionic type, and ionic strength. Particle size and turbidity gradually decreased with the increase of temperature from 50°C to 90°C. Higher proportion of protein was more favorable for the formation of β‐Lg‐GA complex. When mass ratio of GA /β‐Lg was greater than or equal to 2:1, GA was excessive and saturated by adsorption. Heating not only denatured β‐Lg, but also promoted structural rearrangement of protein and polysaccharide to form a more stable core–shell structure. In the heat‐treated β‐Lg‐GA composite system, polysaccharide played an important role. The formation of the nanoparticles relied on thermal denaturation and aggregation of protein, electrostatic, hydrophobic, and hydrogen bonding interactions between β‐Lg and GA were also very important. The β‐Lg‐GA nanoparticles formed by heat treatment have higher encapsulation efficiency on EGCG than that of unheated. In the next step, the application of heated β‐Lg‐GA nanoparticles as potential excellent delivery system will be systematically studied.

## CONFLICT OF INTEREST

The authors declare that they have no competing interests.

## ETHICAL APPROVAL

The experiment does not include any animal or human testing.

## Supporting information

Fig S1‐S2

## Data Availability

All data generated or analyzed during this study are included in this article and its supplementary information files.
