# Supplementary material for: Complexation of β‐lactoglobulin with gum arabic: Effect of heat treatment and enhanced encapsulation efficiency
Source: Food Sci Nutr. 2021 Jan 18;9(3):1399–409. doi: 10.1002/fsn3.2103 (PMC7958567; doi:10.1002/fsn3.2103)
Supplement: Supplementary file 1 — Fig S1‐S2 [file FSN3-9-1399-s001.docx]

**Supporting Information**

**Complexation of β-Lactoglobulin with Gum Arabic: Effect of Heat Treatment and enhanced encapsulation efficiency**

## Mengna Cao • Jian Gao • Yang Li • Chengzhi Liu • Jieyu Shi • Fangfang Ni • Gerui Ren^*^ • Hujun Xie

*School of Food Science and Biotechnology, Zhejiang Gongshang University, Hangzhou 310018, People’s Republic of China*

___________________________________________________________________

***Corresponding author: rengerui@mail.zjgsu.edu.cn (G. Ren).

ORCID: Gerui Ren <https://orcid.org/0000-0002-2222-3956>

Fax: +86-571-28008900.

**Effect of different heating time on the particle size of β-Lg-GA complex**

FIGURE S1 Effect of different heating time on the particle size of β-Lg-GA complex at pH = 4.5 (85 °C).

**Effect of different heat treatment temperatures (25-85 °C) on synchronous fluorescence spectra**

FIGURE S2 Effect of different heat treatment temperatures (25-85 °C) on synchronous fluorescence spectra (a) The synchronous fluorescence spectra of β-Lg solution at Δλ = 15 nm; (b) The synchronous fluorescence spectra of β-Lg-GA mixture solution at Δλ = 15 nm; (c) The synchronous fluorescence spectra of β-Lg solution at Δλ = 60 nm; (d) The synchronous fluorescence spectra of β-Lg-GA mixture solution at Δλ = 60 nm.
